# P02-14 KaziBantu ‘healthy schools for healthy communities' - A holistic approach to enhance health literacy and physical activity in primary schools from low-resourced settings in South Africa

**DOI:** 10.1093/eurpub/ckac095.033

**Published:** 2022-08-29

**Authors:** Patricia Arnaiz, Ivan Müller, Danielle Dolley, Larissa Adams, Jan Degen, Nandi Joubert, Siphesihle Nqweniso, Markus Gerber, Rosa Du Randt, Walter Cheryl, Uwe Pühse

**Affiliations:** 1 University of Basel, Basel, Switzerland; 2 Nelson Mandela University, Gqeberha, South Africa

**Keywords:** Quality physical education, health promoting intervention, public primary schools, governmental institutionalization, disadvantaged settings, South Africa

## Abstract

**Backgound:**

The disease profile of low- to middle-income countries is moving towards one seen in Westernised countries, where deaths are mainly attributed to chronic diseases. Children develop risk factors at a young age predisposing them to noncommunicable diseases in adulthood. Most of the risk factors are preventable through healthy lifestyles. Results from South Africa (SA) show that many children, particularly from marginalized communities, do not achieve the minimal requirements of physical activity (PA). Thus, more emphasis needs to be placed on primary prevention strategies, such as incorporating health promotion interventions within established educational and workplace structures. Primary schools present unique opportunities for holistic prevention interventions.

**Methods:**

Using an ecosystem approach, an interprofessional team of PA researchers, public health specialists and digital innovators, together with partners from the ministry of education and ministry of health in SA, was set up to map and tackle the role of physical education (PE) in the SA school system. Experts identified actionable changes at the school, teacher and policy levels. First, a comprehensive health intervention was developed and implemented in primary schools in low resourced settings in the Eastern Cape of SA. The intervention was followed to learn and adapt. Finally, changes in the educational system will be scaled-up and sustained through governmental institutionalization.

**Results:**

In 1994 PE lost its stand-alone subject status and became part of Life Orientation. Ever since, non-specialist teachers lack the confidence and understanding to adequately teach the subject. The interdisciplinary team developed ‘the KaziBantu model (Healthy Schools for Healthy Communities)’, to promote PA and healthy lifestyles in public primary schools through two complementary programs: KaziKidz, a PE toolkit for schoolchildren, and KaziHealth, a workplace health intervention program for teachers. Furthermore, Short Learning Programs have been developed for continued professional development of life orientation teachers, thereby introducing lasting changes within the educational system.

**Discussion/Conclusion:**

PE and health literacy are oftentimes neglected in the SA curriculum, especially in marginalized areas. System-wide changes initiated and sustained through local ownership are critical to ensure long-lasting impact. Our multilateral intervention aimed to achieve this to offer children and teachers a quality education.

